# Effect of PEW and CS on the Thermal, Mechanical, and Shape Memory Properties of UHMWPE

**DOI:** 10.3390/polym12020483

**Published:** 2020-02-21

**Authors:** Run Zhang, Suwei Wang, Jing Tian, Ke Chen, Ping Xue, Yihui Wu, Weimin Chou

**Affiliations:** College of Mechanical and Electrical Engineering, Beijing University of Chemical Technology, Beijing 100029, China; zhangrun_buct@163.com (R.Z.); wangsw90@163.com (S.W.); tianjing_buct@163.com (J.T.); chenke0903@outlook.com (K.C.); wuyh5463@163.com (Y.W.); cwm758452@163.com (W.C.)

**Keywords:** shape memory, UHMWPE, compression molding technology, solid lubricants

## Abstract

Modified ultra-high-molecular-weight polyethylene (UHMWPE) with calcium stearate (CS) and polyethylene wax (PEW) is a feasible method to improve the fluidity of materials because of the tense entanglement network formed by the extremely long molecular chains of UHMWPE, and a modified UHMWPE sheet was fabricated by compression molding technology. A Fourier-transform infrared spectroscopy test found that a new chemical bond was generated at 1097 cm^−1^ in the materials. Besides, further tests on the thermal, thermomechanical, mechanical, and shape memory properties of the samples were also conducted, which indicates that all properties are affected by the dimension and distribution of crystal regions. Moreover, the experimental results indicate that the addition of PEW and CS can effectively improve the mechanical properties. Additionally, the best comprehensive performance of the samples was obtained at the PEW content of 5 wt % and the CS content of 1 wt %. In addition, the effect of temperature on the shape memory properties of the samples was investigated, and the results indicate that the shape fixity ratio (R_f_) and the shape recovery ratio (R_r_) can reach 100% at 115 °C and 79% at 100 °C, respectively, which can contribute to the development of UHMWPE-based shape memory polymers.

## 1. Introduction

Shape memory polymers (SMPs) are considered to be a smart material that can change their shapes from the temporary (named “soft phase”) to the permanent (named “hard phase”) when exposed to external stimuli, such as heat [1], light [2,3,4], pH [5,6], electricity [7], magnetism [8], and biological enzymes [9], and this ability to change shape is called the shape memory effect (SME). SME can be traced back to the “elastic memory” in a US patent on dental materials made of methacrylic ester resin by Vernon in 1941 [10]. The next important milestone in the development of SMPs is the application of heat shrinkable tubes and heat shrinkable films in the 1960s [11,12,13,14]. Nowadays, after decades of development, SMPs have been successfully used in many fields, such as aerospace [15,16,17,18], biomedical [19,20,21,22,23], and smart appliances [24]. Compared with shape memory alloys (SMAs) and shape memory ceramics (SMCs), SMPs have the advantages of lower density, higher strain (up to 800% [25]), excellent processability, good chemical stability, biocompatibility [20,22], and an adjustable biodegradation rate [26]. However, as we all know, the shape recovery stress of SMPs is only 1–10 MPa, while SMAs can reach 400 MPa [27].

Cross-linked polyethylene is widely used as a shape memory material in developed countries, such as low-density polyethylene (LDPE) and high-density polyethylene (HDPE), but the recovery stress can only reach 3 MPa [28], which cannot meet actual needs. This problem can be solved by using ultra-high-molecular-weight polyethylene (UHMWPE). UHMWPE has many excellent properties due to its large molecular chain, such as excellent mechanical and tribological properties, electrical insulation, and biocompatibility, and thus it has been used in chemical, machinery, joint replacements, and other fields [29,30]. However, the research on shape memory of UHMWPE is still in its infancy. Maksimkin believed that the SME of UHMWPE resulted from physical cross-linking formed by extremely long molecular chains [27], which has strong temperature-dependent characteristics [31], and the authors had obtained UHMWPE fiber with an isothermal recovery stress of 22 MPa. Bastiaansen [32] studied the relationship between SME and resting equilibrium viscoelastic properties by mixing UHMWPE and polyethylene (PE). Other researchers adopted chemical cross-linking methods to further improve the SME of UHMWPE. Chen [33,34] used a silane-induced crossing method to mix UHMWPE with a water-carrying agent to prepare a cross-linked UHMWPE with a shape recovery ratio of more than 98%. Takahashi [35] analyzed the mechanism of total hip components prepared by a radiation cross-linking method in response to external strain from the perspective of molecular physics. In addition, smart materials were prepared by blending UHMWPE with metal derivatives by Pucci et al. [36]. Furthermore, researchers have improved the mechanical, thermal, and electrical properties of UHMWPE instead of SME by adding carbon nanotubes (CNTs) and graphene nanoplatelets (GNPs). Reddy [37] studied the dispersion of CNTs in UHMWPE and found that the electrical percolation threshold is 0.05% due to the formation of a two-dimensional conductive network. The same method was used for the study of mixing GNPs with UHMWPE [38]. Lahiri et al. [39] found that the addition of GNPs with a content less than 1% can improve the mechanical properties of the samples, while the biocompatibility of UHMWPE is seriously affected by the content of GNPs. Liu et al. [40] used the modification of GNP surfaces with organosilane to enhance the wear resistance and storage modulus by 980% and 170%, respectively.

We can hardly deny that the technical proposal of the researchers on the SME of UHMWPE is not mature enough, which is mainly because the extremely long molecular chain of UHMWPE forms a dense entanglement network, and thus they could not form a continuous molten phase when the materials were heated above the melting point (T_m_) [41]. However, calcium stearate (CS) can be used as a heat stabilizer of polyvinyl chloride (PVC) and a solid lubricant for processing various plastics, while polyethylene wax (PEW) has excellent cold resistance, heat resistance, chemical resistance, and wear resistance, and can improve the fluidity of polyethylene (PE), polypropylene (PP), and acrylonitrile butadiene styrene (ABS). Thus, CS and PEW were used as solid lubricants during the processing of UHMWPE, which can not only improve the fluidity of the materials [42], but also reduce the probability of the emergence of micro-defects inside the samples [43]. Panin et al. [44] found that the wear resistance increases four times compared with UHMWPE under the condition of dry sliding friction when the amount of CS is 3 wt %, but the mechanical properties do not improve significantly. Zhong et al. [45] prepared self-reinforced all-PE composites by mixing micron-sized UHMWPE and HDPE wax, of which the Young’s modulus, tensile strength, and impact strength of the samples can reach 4.5 GPa, 160 MPa, and 20 kJ/m^2^, respectively. The artificial muscles were manufactured by Maksimkin et al. in the form of coiled UHMWPE fibers with the recovery stress of 27 MPa, and the structural mechanisms were further discussed [46]. Fan and his colleagues [47] utilized the interaction between the partially engaged molecular chains of UHMWPE and the medium crystal phases of PP to realize external stress-free two-way SME of the specimens. Senatov et al. [48] have researched UHMWPE/Al_2_O_3_ nanocomposites as a material for damaged cartilage replacement.

However, few researchers have studied the effect of agents such as antioxidants and plasticizers on shape memory, and no studies have been conducted on the effects of solid lubricants on the SME of UHMWPE. On the background of such a situation, the research on the properties of UHMWPE modified by CS and PEW has both practical value and theoretical value. CS can effectively improve the fluidity of UHMWPE, while PEW has a good compatibility with UHMWPE. Both CS and PEW can significantly reduce the number of product defects. Therefore, the study about the effect of CS and PEW on the crystallinity and the entanglement effect of UHMWPE molecular chains can provide a new research direction for the modification of UHMWPE. In this paper, the effect of the addition of CS and PEW on the mechanical, thermal, and thermomechanical properties were analyzed from the perspective of the molecular chain entanglement and crystallization. Furthermore, the effect of solid lubricants on SME of UHMWPE was also investigated.

## 2. Materials and Methods

### 2.1. Materials

The host polymer matrix used in this study was UHMWPE (GUR 4152) with a density of 0.935 g/cm^3^ and a weight-average molecular weight (M_w_) of 7.8 × 10^6^ g/mol, which was obtained from Celanese (Nanjing) Diversified Chemical Co., Ltd., Nanjing, China. CS powder with a calcium content of 6.5% ± 0.5% and the T_m_ of 147–149 °C was obtained from Meryer Chemical Technology Co., Ltd., Shanghai, China. Besides, PEW (105) powder made of HDPE with the M_w_ of 1500–3500 g/mol, the T_m_ of 100–110 °C, and the density of 0.91 g/cm^3^ was obtained from Multidimensional Chemical Co., Ltd., Shijiazhuang, China.

### 2.2. Sample Preparation

The UHMWPE sheets were prepared by the compression molding technology. Firstly, UHMWPE, PEW, and CS were weighed according to the weight percentage listed in Table 1, and then mixed in a high-speed stirrer (Zhejiang Wuyi Dingcang Daily Metal Products Factory, Wuyi, China) intermittently at 25,000 rpm for about 10 min. Secondly, the mixture with a different content of CS and PEW was molded by the hot press (Zhengzhou Xinhai Machinery Manufacturing Co., Ltd., Zhengzhou, China) at the temperature of 230 °C and the pressure of 4 MPa for 35 min. Subsequently, the mold filled with the melt was transferred to a water-cooled press (Zhengzhou Xinhai Machinery Manufacturing Co., Ltd., Zhengzhou, China) and quenched to room temperature at the pressure of 10 MPa. Finally, the prepared UHMWPE sheets, with a thickness of approximately 4 mm and 2 mm, were cut into the required shapes for testing.

### 2.3. Analytical Methods

Fourier-transform infrared (FTIR) spectroscopy of the samples was tested in the form of sheets (except CS and PEW) fabricated by the compression molding technique. The spectrum of all samples was recorded at room temperature over the range 4000–600 cm^−1^ by an FTIR spectrometer (NEXUS 670, NECO INDUSTRIES INC., Oklahoma, OK, USA) and an attenuated total reflectance (ATR) cell. Besides, the spectra of all samples were averaged over 16 scans with a 4 cm^−1^ resolution.

The crystallinity and melting behavior of samples were tested by a differential scanning calorimeter (DSC 25, TA Instruments, New Castle, DE, USA). Firstly, the sample was heated from 25 to 200 °C in a nitrogen atmosphere at a heating rate of 10 °C /min, and then cooled to 25 °C at the same rate. Besides, the sample was held at 200 °C for 3 min and at 25 °C for 1 min to eliminate thermal history. Then, the process was repeated again, and the curves were recorded. The degree of crystallinity of the sample (*X_c_*) obtained by DSC was calculated through the following equation:

(1) $$X_{C}=\frac{\Delta H_{m}}{\Delta H_{m}^{o}}\times 100\%$$

where Δ*H_m_* is the melting enthalpy of the samples obtained from the DSC test and $\Delta H_{m}^{o}$ is the melting enthalpy of a 100% crystalline sample (for UHMWPE, $\Delta H_{m}^{o}$ = 289 J/g [38]).

Thermal gravimetric analysis (TGA) was carried out to characterize the relationship between weight loss and temperature, and the decomposition and thermal stability of materials. The test was conducted by a TGA instrument (TGA/DSC1, METTLER TOLEDO INSTRUMENTS CO., LTD., Shanghai, China) in a nitrogen atmosphere (20 mL/min) with the temperature range of 25–700 °C at a heating rate of 10 °C/min.

The Vicat softening temperature (VST) is a parameter to evaluate the heat resistance of samples. During the test, the sample with the size of 10 × 10 × 4 mm^3^ was subjected to the force of 10 N with a heating rate of 50 °C/h to obtain a temperature value at which a pressure needle with a size of 1 mm^2^ penetrated the sample to a depth of 1 mm. Each data is the average value obtained by testing 4 samples.

Dynamical mechanical analysis (DMA) is used to measure the entanglement density (υ_e_) and the average molecular weight (M_e_) between the entanglement points [30]. The sample with a size of 35 × 6 × 2 mm^3^ was tested by a single cantilever bending mode of a DMA instrument (DMTA-V, Rheometric Scientific, New Castle, DE, USA). The test sample was scanned from 60 to 170 °C with the heating rate of 3 °C/min and the scanning frequency of 1 Hz. The storage modulus (*E*′) was defined as the rubbery plateau modulus at 160 °C. The *M_e_* was calculated using the following equation:

(2) $$M_{e}=\frac{2\left(1+\upsilon \right)\rho RT}{E^{\prime }}$$

where *ρ* is the density of the materials (for UHMWPE, *ρ* = 0.935 g/cm^3^), *R* is the gas constant, *T* is the absolute temperature, and *υ* is the Poisson ratio (for UHMWPE, *υ* = 0.4 [33]). Additionally, the chain entanglement density (*υ_e_*) was calculated using the following equation:

(3) $$\upsilon_{e}=\frac{\rho }{M_{e}}$$

The mechanical properties of samples were tested by a universal testing machine (KXWW, Chengde Taiding Testing Machine Manufacturing Co., Ltd., Chengde, China) with a load cell of 5 kN. All tensile test samples with the size of 150 × 20 × 4 mm^3^ were tested at a crosshead speed of 50 mm/min to obtain the tensile strength and elongation at break. Besides, the curve diagrams of the relationship between tensile strength and elongation were also recorded. The flexural test samples with the size of 80 × 10 × 4 mm^3^ were tested at a crosshead speed of 10 mm/min to obtain the flexural modulus and flexural strength. Moreover, the notched impact strength of samples was tested with the size of 80 × 10 × 4 mm^3^ at room temperature, and the side of each sample had a standard notch with a depth of 2 mm. In addition, all results of the tensile test and the flexural test were the average of at least four samples, and the results of the notched impact strength were the average of at least eight samples.

**Figure 1:**
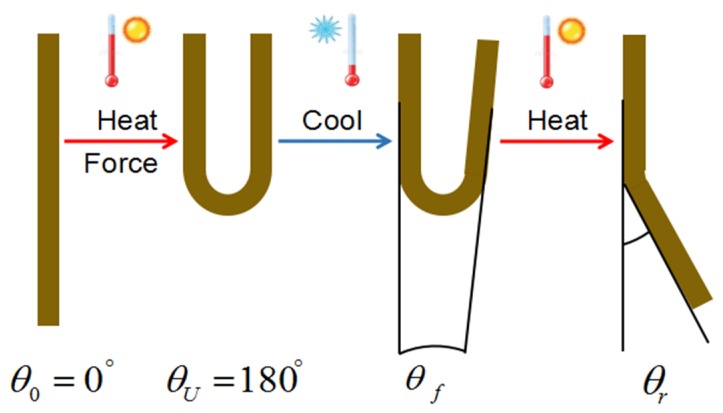
Schematic diagram of the quantitative analysis of shape memory behavior in a bending test.

The bending test was conducted to quantify the shape memory behavior of samples. The schematic diagram of test process is shown in Figure 1. Firstly, a flat sample (θ_0_ = 0°) with the size of 80 × 10 × 2 mm^3^ was heated to the switching temperature (T_SW_) in an oil bath, and then bent into a U-shaped structure with 180° (θ_U_ = 180°). The curvature radius in the tip of the U-shape after 180° bending was 8 mm. Secondly, it was rapidly cooled down to room temperature while the deformation was retained by the external force. Thirdly, after the external force was removed, it was deformed freely in the internal stress field, and then the final fixed angle (θ_f_) was measured by an electronic digital angle ruler (Shengtaixin Technology Co., Ltd., Shenzhen, China) with an accuracy of 0.5°. Finally, it was heated to T_SW_ again without external force, and the final recovery angle (θ_r_) of the sample was recorded. Lastly, the shape fixity ratio (R_f_), the shape recovery ratio (R_r_), and the maximum theoretical stress (σ_max_) could be calculated by the following formula:

(4) $$R_{f}=\frac{\theta_{U}-\theta_{f}}{\theta_{U}}\times 100\%$$

(5) $$R_{r}=\frac{\theta_{U}-\theta_{r}}{\theta_{U}-\theta_{0}}\times 100\%$$

(6) $$\sigma_{max}=E\frac{t}{2R}$$

where *E* is Young’s modulus (for UHMWPE, *E* = 600 MPa [38]), t is the thickness of the sample, and *R* is the curvature radius of the sample.

## 3. Results and Discussion

### 3.1. FTIR Analyses

**Figure 2:**
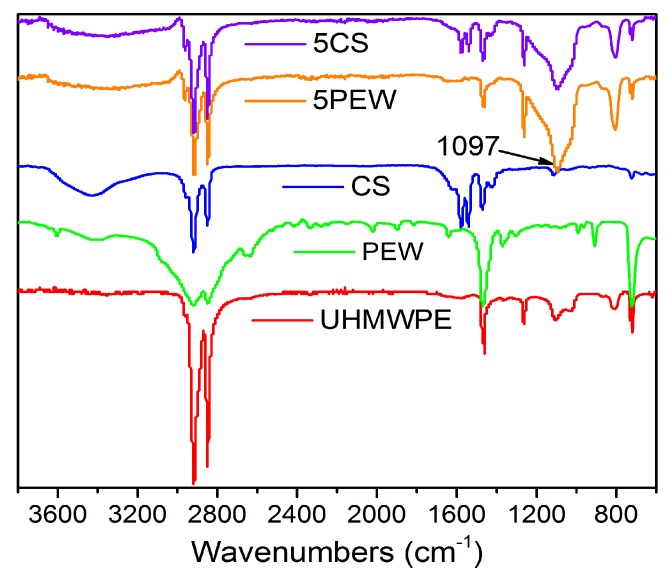
FTIR spectra of UHMWPE, PEW, CS, 5PW, and 5CS.

The FTIR spectra were recorded to characterize the effect of CS and PEW on the molecular structure of UHMWPE, which is shown in Figure 2. It is generally believed that the added PEW and CS played the role of solid lubricants to improve the fluidity of UHMWPE and enhance fusion among materials [42,43]. By comparison, PEW mainly works as the external lubricant, while CS can be used as both external lubricant and internal lubricant. It was found that a new and strong characteristic peak appeared at 1097 cm^−1^ by adding the solid lubricants, which represented the emergence of new ether bonds (C-O-C) inside the materials, and indicated that the addition of solid lubrications changed the molecular structure of UHMWPE during the compression molding process.

### 3.2. Thermal Properties

#### 3.2.1. DSC Analyses

**Figure 3:**
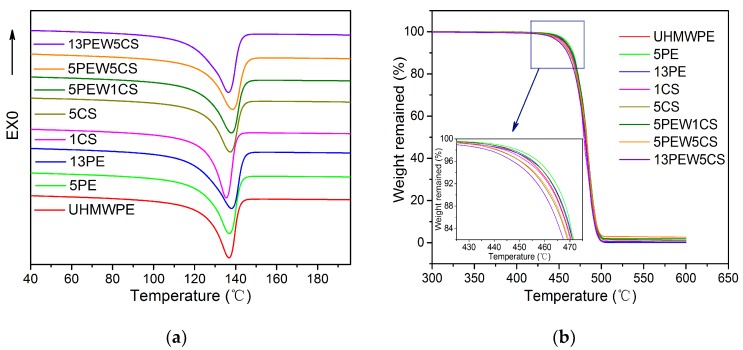
Thermal properties of UHMWPE with different contents of PEW and CS: (**a**) DSC curves; (**b**) TGA curves.

The DSC test of UHMWPE with different contents of PEW and CS was conducted to characterize the crystallization behavior and the initial value (T_m_^on^), maximum (T_m_^max^), and end value (T_m_^off^) of the melting peak of the materials [31]. The DSC curves recorded during the second melting process are shown in Figure 3a, while the degree of crystallinity and the parameter related to the melting peak are listed in Table 2. According to the data in Table 2, the crystallinity of the materials increases with the PEW content and decreases with the CS content, which indicates that the addition of PEW contributes to the growth of the crystal region, while the addition of CS plays the opposite role. Besides, the increased crystallinity of the materials makes the amorphous region smaller, which contributes to the decrease of T_m_^on^, because the melting process starts in the amorphous region. Moreover, the T_m_^on^ of 13PEW5CS is 2.6 °C higher than that of 13PEW. According to the previous studies [38], T_m_^max^ is affected by the micro-defects inside the samples. It can be seen from Table 2 that the T_m_^max^ of the samples gradually increases with the content of PEW and CS, because PEW and CS can penetrate into the gaps between the molecular chains of UHMWPE, reduce the intermolecular force, improve the fluidity of UHMWPE, and reduce the number of micro-defects [43]. However, the addition of PEW with a lower melting point melts first and advances the position of the melting peak, which results in the downward trend of T_m_^max^ from 5PEW5CS to 13PEW5CS.

#### 3.2.2. TGA Analyses

Figure 3b shows the TGA curves of UHMWPE with different contents of PEW and CS, and the parameters such as initial degradation temperature (T_1_) representing 10% weight loss and mid-point degradation temperature (T_50_) representing 50% weight loss are summarized in Table 2. It can be seen that the thermal stability of UHMWPE modified by PEW was improved, while that of CS was declined. Specifically, the T_1_ of materials with the addition of PEW increases from 464.3 to 465.9 °C, while decreasing from 464.3 to 461.9 °C with the addition of CS, which is because the molecular chains’ movement of the amorphous region is affected by the crystalline region below the T_m_, and thus results in the hysteresis phenomenon of the degradation process. That means that the larger the crystalline region, the less likely the movement of molecule chains. However, when PEW and CS are applied to modify UHMWPE simultaneously, the thermal stability of materials declines. According to the above analysis, it can be seen that there is physical cross-linking between the molecular chains in the amorphous region, but it cannot fundamentally hinder the movement of the molecular chains, while the crystal regions can hinder the movement seriously.

#### 3.2.3. VST Analyses

**Figure 4:**
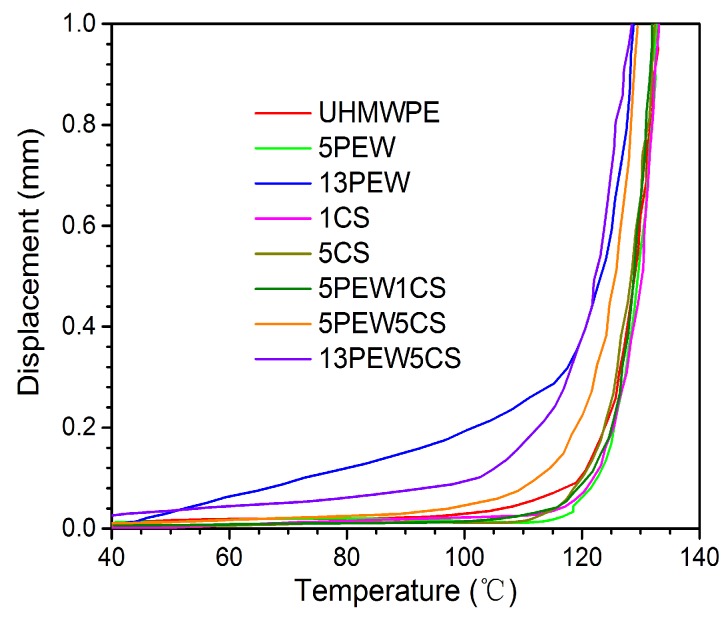
The Vicat softening temperature (VST) curves of UHMWPE with different contents of PEW and CS.

Figure 4 shows the effect of PEW and CS on the VST of the samples. The VST curve is closely related to the T_m_ of the materials. According to the curves in Figure 4, the addition of CS shows little effect on the VST curves of the samples, while the effect of PEW on the VST curves is more and more significant with the increase of PEW content. By comparison, the VST changes little at 5PEW, but decreases from 132.4 to 127.4 °C with the further increase of PEW content, which is mainly because PEW with a lower T_m_ melts first and destroys the structure of the samples. In addition, comparing 13PEW5CS with 13PEW, it can be found that the former curve has an obvious plateau area below 90 °C, while the latter does not.

### 3.3. Thermomechanical Properties

**Figure 5:**
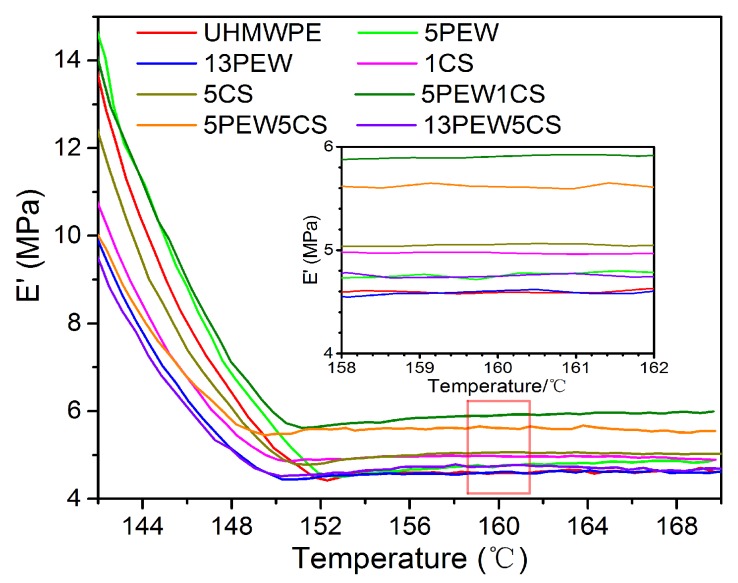
Storage modulus–temperature curves of UHMWPE with different contents of PEW and CS.

The DMA test is considered to be an effective method to measure the molecular weight between physically effective cross-linking points, including physical entanglement and chemical cross-linking [30]. The DMA curves of UHMWPE with different contents of PEW and CS are shown in Figure 5, and the platform modulus (E′), the average molecular weight between entanglement points (M_e_), and the entanglement density (υ_e_) are summarized in Table 3. The platform modulus, which is also known as the storage modulus in the rubbery plateau, is a function of entanglement and cross-linking. Larger storage modulus generally results in greater entanglement density. 

It is not difficult to see that E′ of UHMWPE modified by either PEW or CS has a little change, while the E′ of UHMWPE modified by PEW and CS increases first and then decreases with the increase of content. According to the υ_e_ calculated by the E′ in Table 3, the υ_e_ of UHMWPE that is modified by CS can reach the maximum of 499 mol/m^3^, while the PEW is only about 469 mol/m^3^, which indicates that the effect of CS on physical entanglement of UHMWPE molecular chains is more significant than that of PEW. Meanwhile, the excessive content of PEW results in the decrease of the υ_e_. Obviously, the physical entanglement is more significantly enhanced when UHMWPE is modified by PEW and CS simultaneously, and the maximum υ_e_ can reach 583 mol/m^3^ of 5PEW1CS. Both PEW and CS can improve the movement capacity of UHMWPE chains without external force, which results in the more disordered the molecular chain, the more physical entanglement points and the greater the entropy. However, PEW with a smaller molecular weight can enter the gaps among the molecular chains of UHMWPE during the compression molding process, and the excessive content of PEW occupies the movement space of UHMWPE chains and hinders the movement of UHMWPE chains, which reduces the υ_e_ of materials.

### 3.4. Mechanical Properties

#### 3.4.1. Tensile Test Analyses

**Figure 6:**
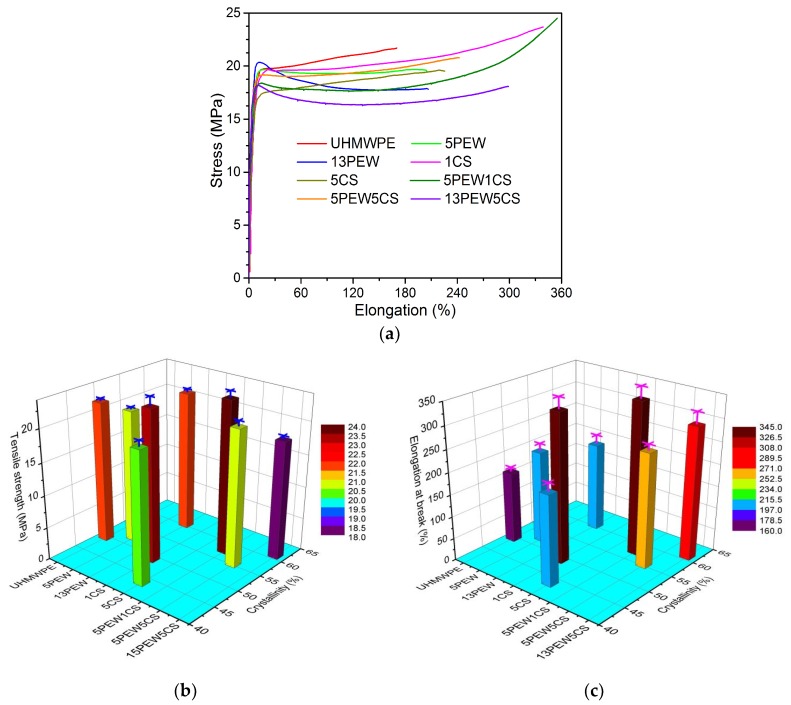
Tensile tests of the samples: (**a**) representative stress–elongation curves; (**b**) tensile strength; (**c**) elongation at break.

**Figure 7:**
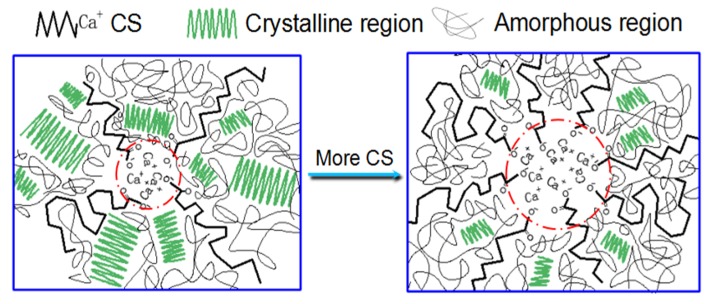
Schematic representation of the effect of CS addition to UHMWPE. This figure highlights the possible ways in which the increase in the size of calcium ionic clusters reduces their strength.

The representative stress–elongation curves obtained in the tensile experiments are shown in Figure 6a, and the tensile strength and the elongation at break of the samples with different contents of PEW and CS are shown in Figure 6b,c, respectively. The tensile strength listed in Figure 6b refers to the maximum tensile strength during the stretching process, including the yield strength for 13PEW or the fracture strength. It can be found from Figure 6 that PEW and CS can improve the elongation at break of the materials, and make the samples have an obvious plastic deformation process in the tensile test. Obviously, pure UHMWPE shows deformation of high elasticity and no plastic deformation, which indicates that pure UHMWPE chains may only have segment movements of the molecular chains during the stretching process. However, significant slippage emerges in molecular chains or crystal regions due to the lubrication of PEW and CS, especially for the samples simultaneously modified by PEW and CS. Besides, the elongation at break of the samples modified by PEW is smaller than that of the samples modified by CS, which is because the former mainly emerges as a slippage between crystal regions, while the latter mainly emerges as a slippage between molecular chains. In addition, the slippage caused by the external force results in the orientation of the molecular chains, which improves the tensile strength from 21.6 MPa of UHMWPE to 23.5 MPa of 1CS, as shown in Figure 6b, and the break caused by the slippage of the crystal regions makes the fracture strength less than the yield strength. Furthermore, the tensile strength decreases from 23.23 MPa of 1CS to 20.1 MPa of 5CS because the increased size of calcium ionic clusters may hinder the slippage of the molecular chains [49]. The schematic diagram describing the increased size of the calcium ionic clusters is shown in Figure 7. In addition, the simultaneous addition of PEW and CS makes the samples show better plastic deformation and higher mechanical properties, such as 5PEW1CS with the elongation of 344% and tensile strength of 23.6 MPa. However, the excessive content of PEW and CS can decrease the elongation at break and tensile strength to 299% and 18.2 MPa, respectively, such as 13PEW5CS, which is mainly due to the excessive slippage of molecular chains caused by the solid lubricants, resulting in the absence of molecular chain orientation.

#### 3.4.2. Three-Point Bending Test Analyses

**Figure 8:**
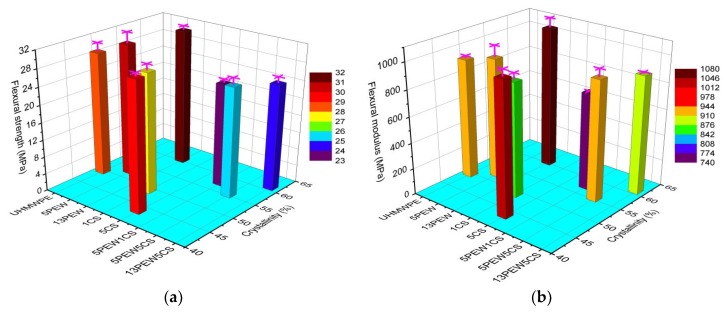

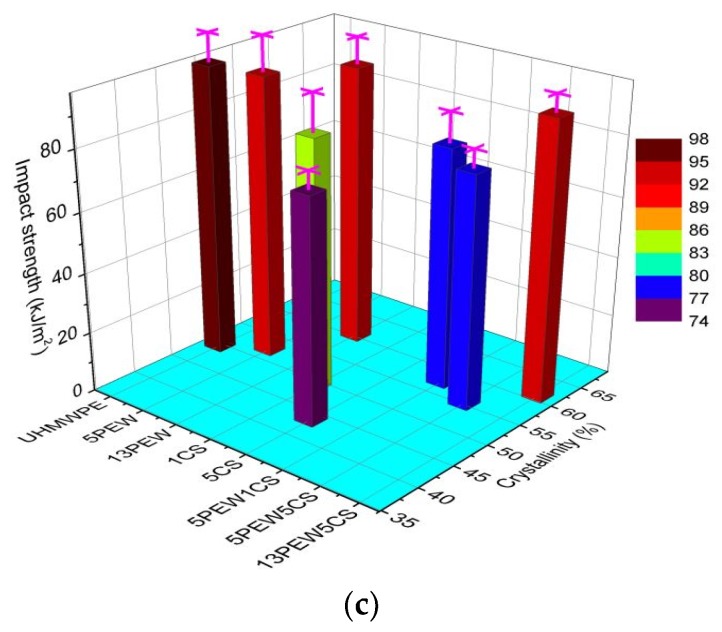
Mechanical properties of the samples: (**a**) flexural strength; (**b**) flexural modulus; (**c**) notched impact strength.

Flexural strength refers to the ability of a material to resist bending. Flexural strength and flexural modulus of the samples are shown in Figure 8a,b, respectively. The flexural strength is significantly affected by the crystallite size and crystallinity of the samples. The flexural strength and flexural modulus increase with the PEW content, and first increase and then decrease with the CS content, while the flexural strength of UHMWPE modified by PEW and CS decreases significantly. It was found that the flexural strength from 28.7 to 31.6 MPa and the flexural modulus from 919.1 to 1079.9 MPa of the samples modified by PEW increase with the increased crystallinity, while the samples modified by CS show the opposite trend. Compared with 1CS, the higher flexural strength and flexural modulus of 5CS attributes to the fact that the increased size of calcium ionic clusters hinders the movement of molecular chains, as shown in Figure 7. Besides, when PEW and CS are applied to modify UHMWPE simultaneously, the added CS could decline the flexural performance of the samples. For example, although 13PEW and 13PEW5CS have almost the same crystallinity, the flexural strength and flexural modulus of the former are higher than those of the latter.

#### 3.4.3. Notched Impact Test Analyses

The notched impact strength of the samples with different contents of PEW and CS is shown in Figure 8c. Compared with pure UHMWPE, the notched impact strength of samples with the addition of PEW and CS decreases from 96.5 to 74.8 kJ/m^2^, which is mainly due to the fact that PEW and CS can penetrate into the gaps between the molecular chains of UHMWPE and change the molecular weight distribution.

### 3.5. Shape Memory Behaviors

**Figure 9:**
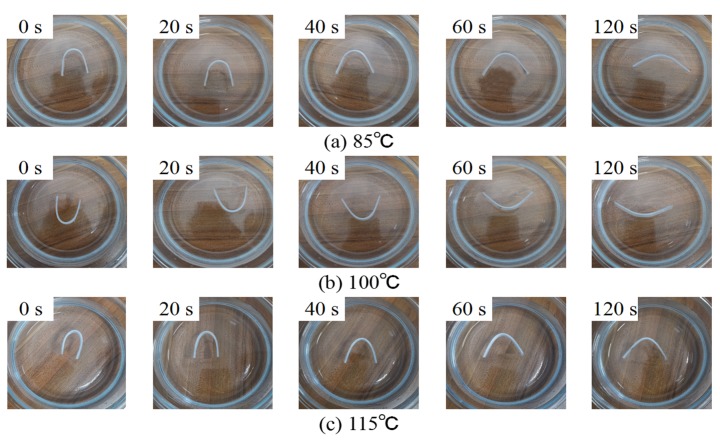
Representative shape recovery processes of 13PEW5CS over time at different T_SW_: (**a**) T_SW_ = 85 °C; (**b**) T_SW_ = 100 °C; (**c**) T_SW_ = 115 °C.

**Figure 10:**
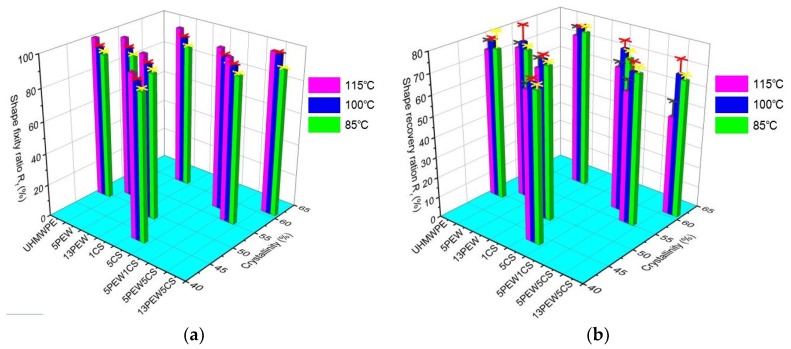
Shape memory properties of the samples: (**a**) shape fixity ratio; (**b**) shape recovery ratio.

Most SMPs contain two parts inside, including the “hard phase” and the “soft phase”. The hard phase mainly plays a fixity role to maintain a permanent shape, while the soft phase mainly plays a deformation role to provide the SMPs with a temporary shape [14]. As shown in Table 2, the crystalline region could not melt at T_SW_ in this study. Therefore, the crystalline regions in UHMWPE mainly acted as the “hard phase”, while the amorphous regions acted as the “soft phase”. According to Equation (6), the maximum theoretical stress is 75 MPa. Figure 9 shows the representative shape recovery process of 13PEW5CS over time at different T_SW_, which indicates that the SME has a strong temperature dependence [31]. Figure 10 shows the R_f_ and the R_r_ of UHMWPE modified by PEW and CS within two minutes (little shape recovery at more than two minutes) at different T_SW_. It can be seen that the R_f_ remains basically constant at the same T_SW_, but increases with the temperature because of more movement of the chain segments in the amorphous region, such as 90% at 85 °C, 95% at 100 °C, and 100% at 115 °C, which is consistent with the research of Wu et al. [50]. In particular, the R_f_ of 13PEW5CS reaches 99% at 100 °C. On one hand, a continuous “hard phase” cannot be formed to resist deformation; on the other hand, the deformation of amorphous phases among the crystal regions also drives the deformation of crystal regions.

The shape recovery process is the process of releasing the energy stored during the deformation at T_SW_ [51]. Compared with the maximum value (approximately 77%) of R_r_ of UHMWPE at 100 °C, it can be seen from Figure 10b that the R_r_ of each group sample ranges from 48% of 13PEW5CS at 115 °C to 79% of 13PEW at 100 °C, which indicates that the addition of PEW and CS does not significantly improve the R_r_ of UHMWPE. In addition, the R_r_ of the samples increases first and then decreases with the increase of the T_SW_, which may be attributed to the fact that the energy has already released as the external force is removed at low temperature, and the movement of the chain segments dissipates part of the energy at high temperature. It is also found that the R_r_ increases with the increased crystallinity due to the greater recovery stress that emerges when the external force coerces the “hard phase” deformation. However, there is no such relationship between R_r_ and crystallinity.

## 4. Conclusions

In this article, the UHMWPE sheets with the shape memory property were prepared by compression molding technology. The FTIR spectra show the generation of chemical bond C-O-C domains at 1097 cm^−1^ in the materials. Further research on the thermal properties of the samples found that the addition of PEW can improve the crystallinity of UHMWPE from 49.6% to 60.3%, while the addition of CS decreases the crystallinity to 43.5%. Besides, the addition of PEW or CS shows the reverse effect on the thermal stability performance because of the effect of crystallization on UHMWPE. However, it should be noted that excessive CS will reduce T_1_ from 464.3 °C of UHMWPE to 460.1 °C of 13PEW5CS with the increased crystallinity. The chain entanglement density can be significantly improved from 453 mol/m^3^ of UHMWPE to 583 mol/m^3^ of 5PEW1CS. Due to the increased degree of slippage of the UHMWPE molecular chains by the addition of PEW and CS, modified UHMWPE exhibits obvious plastic deformation, which further improves the tensile strength from 21.7 MPa of UHMWPE to 23.6 MPa of 5PEW1CS and elongation at break from 161.6% of UHMWPE to 344.4% of 5PEW1CS. The temperature dependence of shape memory was characterized and found that the R_f_ of modified UHMWPE increases with the temperature and reaches 100% at 115 °C, but the value of R_r_ is generally low, and the maximum is just 79%, therefore further research is required to be focused on the improvement of R_r_.

## Figures and Tables

**Table 1:** The formulation of ultra-high-molecular-weight polyethylene (UHMWPE)/polyethylene wax (PEW)/calcium stearate (CS) materials.

		Content (wt %)							
		UHMWPE	5PEW	13PEW	1CS	5CS	5PEW1CS	5PEW5CS	13PEW5CS
UHMWPE		100	95	87	99	95	94	90	82
PEW		--	5	13	--	--	5	5	13
CS		--	--	--	1	5	1	5	5

**Table 2:** Characteristic points of the differential scanning calorimeter (DSC) and TGA for UHMWPE with different contents of PEW and CS.

Sample	DSC				TGA		
	T_m_^on^ (°C)	T_m_^max^ (°C)	T_m_^off^ (°C)	Crystallinity (%)	T_1_ (°C)	T_50_ (°C)	Ash Content (%)
UHMWPE	125.3	136.9	141.9	49.62	464.3	480.7	0.256
5PEW	122.9	136.9	142.3	52.16	466.2	481.6	0.013
13PEW	120.2	138.1	144.3	60.29	465.9	481.1	0.105
1CS	125.1	136.4	141.3	49.08	463.9	479.4	0.352
5CS	124.5	137.4	143.3	43.51	461.9	481.8	0.988
5PEW1CS	123.9	137.9	144.1	57.52	465.1	482.7	1.814
5PEW5CS	123.4	138.3	144.9	55.24	462.6	482.6	2.586
13PEW5CS	122.8	137.5	143.0	60.96	460.1	480.6	0.331

**Table 3:** E′, M_e_, and υ_e_ values of UHMWPE with different contents of PEW and CS.

		UHMWPE	5PEW	13PEW	1CS	5CS	5PEW1CS	5PEW5CS	13PEW5CS
	
E′ (MPa)		4.587	4.755	4.603	4.971	5.060	5.907	5.607	4.761
M_e_ (g/mol)		2066	1993	2059	1907	1873	1605	1690	1991
υ_e_ (mol/m^3^)		453	469	454	490	499	583	553	470
